# The Influence of Solvent, Host, and Phenological Stage on the Yield, Chemical Composition, and Antidiabetic and Antioxidant Properties of *Phragmanthera capitata* (Sprengel) S. Balle

**DOI:** 10.1155/2020/6284925

**Published:** 2020-11-18

**Authors:** Césaire Feudjio, Muhammad Arfat Yameen, Guy Sedar Singor Njateng, Muhammed Ahsan Khan, Stephen Lacmata Tamekou, James Deke Simo Mpetga, Jules-Roger Kuiate

**Affiliations:** ^1^Research Unit of Microbiology and Antimicrobial Substances, Faculty of Science, Department of Biochemistry, University of Dschang, P.O. Box 67, Dschang, Cameroon; ^2^COMSATS University of Information Technology, Abbottabad Campus, Islamabad 22060, Pakistan; ^3^Natural Products Chemistry Research Unit, Faculty of Science, Department of Chemistry, University of Dschang, P.O. Box 67, Dschang, Cameroon

## Abstract

*Phragmanthera capitata* was reported to possess many biological properties making it a good candidate for the formulation of a phytomedicine with multiple effects. In this work, we studied some factors likely to modify these therapeutic properties with the aim to contribute to its standardization as an improved traditional medicine. *P. capitata* parasitizing *Persea americana, Psidium guajava,* and *Podocarpus mannii* were harvested at three phenological stages (vegetative, flowering, and fruiting stages). The extracts were prepared by maceration in n-hexane, ethyl acetate, ethanol, methanol, and distilled water. The total phenolic, flavonoid, flavonol, and tannin contents were measured using appropriate methods. The antioxidant potential of extracts was investigated using TAC, DPPH scavenging, and FRAP methods. The *α*-amylase and *α*-glucosidase inhibitory activities of extracts were determined using enzymatic methods. The ethyl acetate extracts with the best phenolic content were subjected to HPLC analysis. The extraction yields were higher with methanol. The ethyl acetate extract of *P*. *capitata* harvested from *P. guajava* showed a stable HPLC profile during the development of the plant, while extracts from the plant collected from *P. americana* and *P. mannii* showed both qualitative and quantitative variations according to phonological stages of the plant. The inhibition of *α*-amylase was more pronounced for *P. capitata* harvested from *P. guajava*, decreasing during flowering and fruiting, while inhibition of *α*-glucosidase was not influenced by the phenological stage and the host of the plant. The *α*-amylase inhibitors were better extracted by ethyl acetate and those of *α*-glucosidase by ethanol or methanol. The phenolic contents and antioxidant properties of the extracts were influenced by the phenological stage of *P. capitata* and its hosts. These results suggest that it is preferable to harvest *P. capitata* during flowering or during fruiting stages on any host. None of the used solvents permitted an optimal extraction of active principles form *P*. *capitata*, suggesting that the mixture of solvents must be considered in further studies.

## 1. Introduction

*Phragmanthera capitata* is a mistletoe plant belonging to the Loranthaceae family. It is a mandatory hemiparasite that attaches and enters the stems and branches of its host tree through a haustorium [1, 2]. This plant is widely distributed in Cameroon and in some other African countries. It is characterized by yellow flowers with red apex [3]. It can grow on various plants, including *Persea americana*, *Psidium guajava*, *Citrus sinensis*, *Podocarpus mannii*, *Hevea brasiliensis*, *Spondias mangifera*, *Garcinia kola*, *Manniophyton fulvum*, *Theobroma cacao*, and *Citrus* spp. [4]. In Cameroon, leaf, stem, or whole plant is claimed to cure up to 38 diseases, including infectious diseases, nerve pain, cancers, and cardiac disorders, but mostly used to cure hypertension and diabetes [5, 6].

Previous scientific reports demonstrated that extracts from this plant have many biological activities, such as antioxidant, anti-inflammatory [7], hematopoietic potentiating [8], antibacterial, antifungal [9], antisecretory, gastroprotective, and antiulcer [10], and that they also enhance steroidogenetic and spermatogenetic activities [11]. In a previous research work in our laboratory, we demonstrated that the administration of *Phragmanthera capitata* aqueous extract to diabetic rats significantly decreased hyperglycaemia at the dose used by the herbal medicine doctors. It showed hypolipidemic and immunomodulatory effects as well [12]. Furthermore, the water extract from *P. capitata* showed no toxic reactions, no change in behavior, and no mortality at a dose of 3000 mg/kg and 5000 mg/kg in mice and Wistar rats [7, 13], while acetone, methanol, ethanol, and water extracts showed not toxic (lethal concentration > 1 mg/mL) in Brine shrimp hatchability assay. Hence, the solvent extracts from this plant can be further explored for the development of plant-based pharmaceuticals drugs [14].

This parasitic plant is a real problem for crops. Thus, several methods using synthetic herbicides have been developed to eradicate it without much success. Chemical control has side effects on the environmental pollution and food health [15]. Given its multiple biological properties, this plant could be exploited as a phytomedicine and deprived of its host for this reason. In addition, one of the major problems faced by the phytomedicine is the unavailability of rigid quality control profiles for herbal materials and their formulations [16]. Moreover, the solvent effect is an important parameter for phytomedicine standardization, allowing selection of a suitable solvent for herbal processing. The studies of Ohikhena et al. [17, 18] showed the influence of the solvent on the antioxidant and antidiabetic activities of the extract of leaves from *P. capitata* growing on rubber tree. The standardization of extracts is necessary to guarantee the desired properties at all times. Indeed, plant extract composition and biological activities may vary due to climatic differences, phenological stages, soil composition, and environmental stress. Additionally, for some plants, chemo varieties and chemo cultivars may exist [14], making it difficult for untrained people to discriminate against them. The harvesting, drying, storage, transportation, and processing methods may also influence herbal quality and pharmacological properties [19].

The present work aimed to study the influence of certain factors, in particular, the phenology of the plant, the extraction solvent, and the nature of the host of *P. capitata* on the phytochemistry, antioxidant, and antidiabetic properties of the extracts of this parasitic plant. This study should generate preliminary data for a possible standardization of extracts of *P. capitata* as an antioxidant phytomedicine to fight oxidative stress associated with the many disease conditions that are currently being managed locally with the plant.

## 2. Materials and Methods

### 2.1. Processing of Plant Samples

*Phragmanthera capitata* (Sprengel) S. Balle was harvested in Bamendou village, Menoua division of the west region of Cameroon. It was recommended by a traditional healer working in the locality. The whole plant sample was harvested in the morning, between 8 and 10 am, from each of three different plant hosts, namely, *Persea americana*, *Psidium guajava,* and *Podocarpus mannii*, and at three different phenological stages: during vegetative (January 2018), during flowering (March 2018), and during fruiting (April 2018). *P. capitata* and its selected host plants were authentified at the Cameroon National Herbarium in Yaoundé, by comparison to registered voucher specimens (Table 1). Each plant sample was cleaned and dried for 3 weeks in a ventilated room at ambient temperature (22 ± 2°C). The dried plant material was ground and stored in plastic bags until extraction. The study was conducted according to the Guidelines on the Conservation of Medicinal Plants of the WHO [20].

### 2.2. Preparation of Extracts

Five different solvents of increasing polarity were individually used for the preparation of extracts, including n-hexane, ethyl acetate, ethanol, methanol, and water. One hundred grams (100 g) of each powder was macerated in 500 mL in the corresponding solvent for 48 h under mechanical stirring. The resulting mixture was vacuum filtered through Whatman filter paper N 1. The obtained organic filtrates (except aqueous filtrate) were evaporated under low pressure using a Buchi R210 evaporator at 40°C. The resulting extracts were subjected to a 40°C drying in an oven for 24 hours to remove the residual solvent. The aqueous filtrates aliquots of 20 ml were dried in an oven at 40°C for 5 days in stainless plates (30 cm diameter).

### 2.3. Evaluation of Antidiabetic Activity of *P*. *capitata* Extracts

#### 2.3.1. Alpha-Amylase Inhibitory Assay

The *α*-amylase inhibitory effects of extracts were determined using the DNSA method as described by Kazeem et al. [21] adapted for 96-well microplates. The DNSA reagent was freshly prepared by dissolving 1 g of DNSA and 30 g of potassium sodium tartrate in 40 mL of distilled water, after which 20 *μ*L of NaOH (2 M) was added, and then, the total volume was adjusted to 100 mL with distilled water. This was performed under magnetic stirring on a 40°C hot plate. Aliquots of 20 *μ*L of sodium phosphate buffer (0.02 M, pH 6.9) and *α*-amylase solution (0.5 mg/mL) were added to 20 *μ*L of varying concentrations (3.25–800 *μ*g/mL) of the extract or acarbose (2 mg/mL in DMSO 1%). The microplate was preincubated at 37°C for 5 min, after which 20 *μ*L of 1% starch solution in sodium phosphate buffer (0.02 M, pH 6.9) was added and then further incubated at 37°C for 30 min. The reaction mixture was stopped by adding 20 *μ*L of DNSA reagent. The microplate was then placed in a waterbath at 85°C for 10 min to allow the reaction between DNSA and maltose from the hydrolysis of starch. This reaction was then stopped by placing the microplate in an ice waterbath for 3 min. The reaction mixture was diluted with 100 *μ*L of distilled water. A negative control was prepared using the same procedure replacing the extract with DMSO solution (1%). The absorbances were read at 540 nm using an Elisa Bio-Rad PR 4100 microplate reader. The *α*-amylase inhibitory activity was calculated as percentage inhibition of starch hydrolysis:

(1) $$\begin{array}{c} \%\text{ inhibition}=\left[\frac{{\text{Absorbance}}_{\text{control }}-{\text{absorbance}}_{\text{test }}}{{\text{Absorbance}}_{\text{control }}}\right]\times 100. \end{array}$$

Concentrations of extracts or acarbose resulting in 50% inhibition of enzyme activity (IC_50_ in *μ*g/mL) were obtained by applying regression analysis.

#### 2.3.2. Alpha-Glucosidase Inhibitory Assay

The *α*-glucosidase inhibitory activities of extracts were determined according to the method described by Bljajić et al. [22] adapted for 96-well microplates, using *α*-glucosidase from *Saccharomyces cerevisiae* type I (Sigma-Aldrich, US) and p-nitrophenylglucopyranoside (pNPG, Sigma-Aldrich, US) as the substrate. The solution of *α*-glucosidase was prepared at 0.5 U/mL by dilution of a 9 U/mL stock solution. The substrate solution was prepared by dissolving 3 mg of pNPG in 1 mL of phosphate buffer (0.07 M, pH 6.8). Aliquots of 62 *μ*L of phosphate buffer and 12 *μ*L of *α*-glucosidase solution were added to 10 *μ*L of varying concentrations (3.25–800 *μ*g/mL) of the extract or acarbose (2 mg/mL in DMSO 1%). The microplate was preincubated at 37°C for 5 min. Then, 16 *μ*L of substrate (pNPG) solution was added to start the reaction. The microplate was homogenized and incubated at 37°C for 30 min. A negative control was prepared using the same procedure replacing the extract with DMSO solution (1%). The absorbances were read at 405 nm using an ELx800 microplate reader. Percentage of enzyme inhibition by the extract (or acarbose) was calculated using the following equation:

(2) $$\begin{array}{c} \%\text{ inhibition}=100-\left[\frac{{\text{Absorbance}}_{30\min}-{\text{absorbance}}_{5\min}}{{\text{Absorbance}}_{\text{control}}}\right]\times 100. \end{array}$$

Concentrations of extracts or acarbose necessary to inhibit 50% activity of the enzyme (IC_50_) were obtained by applying regression analysis.

### 2.4. Chemical Characterization and Antioxidant Properties of *P. capitata* Extracts

Five known compounds were isolated and characterized in ethyl acetate extracts, and this is described in the Supplementary material. The other aspect of chemical characterization of different extracts comprised total phenol (TPC), total flavonoids (TFC), total flavonols (TfnC), and total tannin content (TTC) determinations. For this purpose, a stock solution of each plant extract was prepared at a concentration of 4 mg/mL in DMSO 1%. Prior to this, the ethyl acetate extract that showed the best antioxidant activity was submitted to HPLC for compound identification.

#### 2.4.1. HPLC Profile of Ethyl Acetate Extracts

HPLC analyses were performed on different ethyl acetate extracts. It was used to determine the gallic acid, quercetin, rutin, and tannic acid proportions in the ethyl acetate plant extracts. The HPLC system used (Shimadzu 20 AD, Japan) consists of an ultraviolet detector, a binary pump, a 20 *μ*L injection loop, and Shim-Pack GIST C18 (150 mm × 4, 6 mm i.d. × 5 *μ*m) column. The mobile phase consisted of two solvents, namely, acetonitrile, HPLC grade (solvent A), and formic acid (1% in deionized water), HPLC grade (solvent B). The elution gradient was established as follows: 0 min (5% A and 95% B), 0–21 min (5–20% A and 95–80% B), 21–30 min (20–25% A and 80–75% B), 30–32 min (25–100% A and 75–0% B), 32–39 min (100–100% A and 0% B), 39–40 min (100–5% A and 0–95% B), and 40–45 min (5–5% A and 95% B). The flow rate of this mobile phase was 0.7 mL/min.

The extracts and standard phenolic compounds were prepared at a concentration of 10 mg/ml in acetonitrile (HPLC grade). Each solution was then diluted to 100 *μ*g/mL and then filtered with a syringe filter (Corning, 0.45 *μ*m) to obtain the solutions to be analyzed. The mixture of standards phenolic compounds was prepared immediately, and 20 *μ*L of mixture solution was injected into HPLC to get the profile of standard. After column cleaning, 20 *μ*L of each plant extract was then injected into the HPLC system to determine its chemical profile. The proportion of gallic acid, quercetin, rutin, and tannic acid in the ethyl acetate plant extracts was determined by comparing the peak area (AUC) of the compound identified in the profile of the extract to its area in the profile of the standard [23].

#### 2.4.2. Total Phenolic Content Determination

The total phenolic content (TPC) of extracts was determined using the Folin–Ciocalteu colorimetric method as described by Horszwald and Andlauer [24] adapted for 96-well microplates. A calibration curve was established with gallic acid (0–50 *μ*g/mL). The Folin-Ciocalteu reagent (90 *μ*L) (Sigma) diluted 10 times with distilled water was mixed to 20 *μ*L of the extract or gallic acid solution. A 7% Na_2_CO_3_ solution (90 *μ*L) was then added followed by thorough mixing and incubation in the dark at room temperature (22 ± 2°C) for 60 min. The blank solution was prepared as above, replacing the extract or gallic acid with distilled water. The absorbance was read at 765 nm using an ELx800 microplate reader. The amount of phenolic content was derived from the calibration curve and expressed as mg equivalent of gallic acid per gram of dry extract (mg GAE/g).

#### 2.4.3. Total Flavonoids Content

The total flavonoids content (TFC) was assayed using the AlCl_3_ method as described by Laloo and Sahu [25] adapted for 96-well microplates. A calibration curve was established with rutin (0–100 *μ*g/mL). The AlCl_3_ solution (10 *μ*L) (10% in distilled water) was added to 20 *μ*L of the extract or rutin solution. After 5 min, 10 *μ*l of sodium acetate solution (1% in distilled water) was added to each well. The total volumes were adjusted to 200 *μ*L with distilled water, followed by thorough mixing and incubation in the dark at room temperature (22 ± 2°C) for 15 min. The blank solution was prepared as above, replacing the extract or rutin by distilled water. The absorbance was read at 415 nm using an ELx800 microplate reader. The amount of flavonoids in plant extracts was derived from the calibration curve. Flavonoid content was expressed in mg equivalents of rutin per g of dry extract (mg RUE/g).

#### 2.4.4. Total Flavonols Content

The total flavonols content (TfnC) was assayed using the AlCl_3_ method according to Awah et al. [26] adapted for a 96-well microplate format. The calibration curve was established with rutin (0–100 *μ*g/mL). Briefly, 40 *μ*L of the plant extract or rutin solution was mixed with 40 *μ*L AlCl_3_ (20 mg/mL in ethanol, HPLC grid). After 5 min, 120 *μ*L of sodium acetate solution (50 mg/mL in ethanol, HPLC grade) was added to each well. The blank was prepared by replacing the extract or rutin with distilled water. The absorbance of each mixture was read at 440 nm after 2 h 30 min of incubation in the dark and at room temperature, using an ELx800 microplate reader. The amount of flavonols in plant extracts was derived from the calibration curve and expressed in mg equivalents of rutin per gram of dry extract (mg RUE/g).

#### 2.4.5. Total Tannin Contents

Total tannin contents (TTC) were assayed according to the Gupta and Verma [27] protocol adapted for 96-well microplates. The calibration curve was established with tannic acid (0–100 *μ*g/mL). Briefly, 20 *μ*L of 0.1 M FeCl_3_ (in 0.1 N HCl) was added to 20 *μ*L of the plant extract or tannic acid, followed immediately by addition of 20 *μ*L of 0.008 M of K_3_Fe(CN)_6_ (in distilled water). The volumes were adjusted to 200 *μ*L with 140 *μ*L of distilled water followed by thorough mixing and incubation in the dark room temperature for 10 min to allow color to become more stable. The blank was prepared by replacing the extract or tannic acid with distilled water. The absorbances were read at 720 nm using an ELx800 microplate reader. The amount of tannin in the plant extracts was derived from the calibration curve and expressed as mg of tannic acid equivalents per g of dry extract (mg TAE/g).

*(1) Determination of Antioxidant Activities*. Three methods were used to determine the antioxidant potential of various extracts: total antioxidant capacity (TAC), ferric reducing antioxidant power (FRAP), and 2,2-diphenyl-1-picrylhydrazyl (DPPH) scavenging activity. For this purpose, a stock solution of each plant extract was prepared at a concentration of 2 mg/mL in DMSO 1%. All experiments were carried out in triplicate.

*(2) Determination of the Total Antioxidant Capacity*. The total antioxidant capacity (TAC) of extracts was measured with the phosphomolybdenum method according to Hossain and Shah [28] protocol adapted for 96-well microplates. The phosphomolybdate reagent solution was prepared freshly by mixing 50 mL of H_2_SO_4_ (0.6 M), 50 mL of Na_3_PO_4_ (28 mM), and 50 mL of (NH_4_)_6_Mo_7_O_24_4H_2_O (4 mM). The calibration curve was established with ascorbic acid (0–50 *μ*g/mL). The phosphomolybdate reagent (150 *μ*L) was added to 15 *μ*L of extracts or ascorbic acid. The blanks were constituted by mixing 150 *μ*L of phosphomolybdate reagent solution and 15 *μ*L of DMSO. The microplate was incubated in a waterbath at 80°C for 90 min, then cooled down to room temperature. The absorbance was read at 695 nm using an ELx800 microplate reader. The total antioxidant capacity (TAC) of plant extracts was derived from the calibration curve of ascorbic acid and expressed as equivalent of ascorbic acid.

*(3) Ferric Reducing Antioxidant Power*. The method of Vijayalakshmi and Ruckmani [29] using K_3_Fe(CN)_6_ adapted on 96-well microplates was used. The calibration curve was established with ascorbic acid (0–100 *μ*g/mL). Volumes of 50 *μ*L of phosphate buffer (20 mM, pH 6.6) and potassium ferricyanide solution (1% w/w) were, respectively, added to 20 *μ*L of the extract or ascorbic acid. The mixtures were incubated in a waterbath at 50°C for 20 minutes. The microplate was removed from the waterbath, and 50 *μ*L of trichloroacetic acid (10% w/w) was added into each well, and then left standing for 60 min. The supernatant (10 *μ*L) was diluted with 90 *μ*L of distilled water, and 10 *μ*L of freshly prepared ferric chloride (0.1% w/w) was added. The reducing power of each well was investigated through the transformation of Fe ^3+^ to Fe ^2+^, and its absorbance was measured at 700 nm using an ELx800 microplate reader. The ferric reducing antioxidant power (FRAP) values of plant extracts were derived from the calibration curve of ascorbic acid and expressed as the equivalent of ascorbic acid.

*(4) DPPH Scavenging Method*. The 2,2-diphenyl-1-picrylhydrazyl (DPPH) radical scavenging activity was determined using the method proposed by Ahmed et al. [30] adapted on 96-well microplates. A solution of DPPH (100 *μ*M) was freshly prepared by dissolving 3.9 mg DPPH in 100 mL of methanol. Aliquots of 180 *μ*L of DPPH solution were added to 20 *μ*L of varying concentrations (3–400 *μ*g/mL) of the extract or ascorbic acid. The microplate was then incubated in the dark for 30 min at room temperature. The blank was prepared as above mentioned without the extract replaced by methanol. Changes in the absorbance of the extract samples were measured at 517 nm using an ELx800 microplate reader. Radical scavenging activities (RSA) were expressed as the inhibition percentage calculated using the following formula:

(3) $$\begin{array}{c} \text{RSA}\left(\%\right)=\frac{{\text{Absorbance}}_{\text{DPPH }}-{\text{absorbance}}_{\text{sample }}}{{\text{Absorbance}}_{\text{DPPH}\cdot }}\times 100. \end{array}$$

The extract concentration corresponding to 50% inhibition (EC_50_) was calculated from the curve of inhibition percentage against the extract concentration.

### 2.5. Statistical Analysis

All assays were performed in triplicate. The data were subjected to analysis of variance, and when differences were observed, the means were compared two by two using the Waller–Duncan test (*p* < 0.05). Each dependent variable (yield, TPC, TFC, TfnC, TTC, TAC, FRAP, DPPH, and IC_50_ of *α*-amylase and *α*-glucosidase inhibition) was compared in the three groups of independent variables (phenological stage of *P. capitata*, host, and extraction solvent). The results were expressed as mean ± standard deviation. Pearson correlation was used to search for the link between the different dependent variables at 5% and 1% thresholds. For these analyses, we used SPSS 25 software for Windows.

## 3. Results

### 3.1. Extraction Yields of *Phragmanthera capitata*

**Figure 1:**
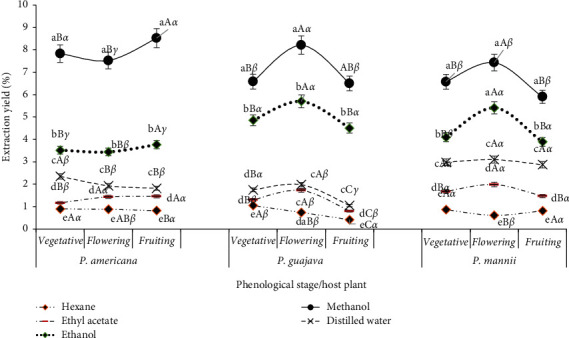
Evolution of the extraction yields of *P. capitata* according to the solvent, phenological stage, and host. a, b, c, d, e: for the same phenological stage and host plant, means carrying different letters are significantly different (*p* < 0.05, Waller–Duncan test); A, B, C: for the same solvent and host plant, means carrying different letters are significantly different (*p* < 0.05, Waller–Duncan test), *α*, *β*, *γ*: for the same solvent and host phenological stage, means carrying different letters are significantly different (*p* < 0.05, Waller–Duncan test).

Extraction yields varied with the solvents used for extraction, the host plant type, and the phenological stage of *P. capitata* (Figure 1). Using methanol as the extracting solvent gave significantly higher (*p* < 0.05) yields compared to using either ethanol, water, ethyl acetate, or hexane, the latter giving the lowest yields. The extraction yield of *P. capitata* harvested on *P. guajava* and *P. mannii* was overall significantly higher (*p* < 0.05) during flowering for an extraction solvent except with hexane. On the other hand, the extraction yield of *P. capitata* from *P. americana* host was not significantly influenced by the phenological stage of *P. capitata*.

### 3.2. HPLC Profile of Ethyl Acetate Extracts

**Figure 2:**
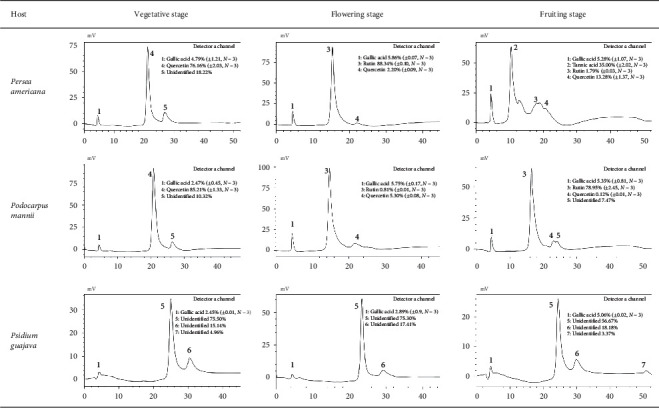
HPLC profile at 280 nm of the ethyl acetate extract of *Phragmanthera capitata* harvested from tree hosts at three different phenological stages.

HPLC profiles of ethyl acetate extracts showed a dynamic accumulation of phenolic compounds in *P. capitata* according to the phenological stage and host plant (Figure 2). The chemical composition of the ethyl acetate extract of *P. capitata* grown on *P. americana* and *P. mannii* was relatively the same before and during flowering. Indeed, during the vegetative stage, quercetin appeared as the main constituent, while during flowering, rutin replaced quercetin as the main constituent. The main differences between these two plant hosts appear during fruiting. At this stage, while tannic acid was highly synthesized in replacement of rutin in *P*. *capitata* growing on *P*. *americana*, rutin remained the most abundant constituent on *P*. *mannii*. Ethyl acetate extract of *P. capitata* collected on *P. guajava* presented a relatively stable chemical composition at the three phenological stages, but very different from what was observed on *P. americana* and *P. mannii*. The main constituent of this extract was not identified.

### 3.3. In *Vitro* Inhibition of *α*-amylase and *α*-glucosidase Activities

**Figure 3:**
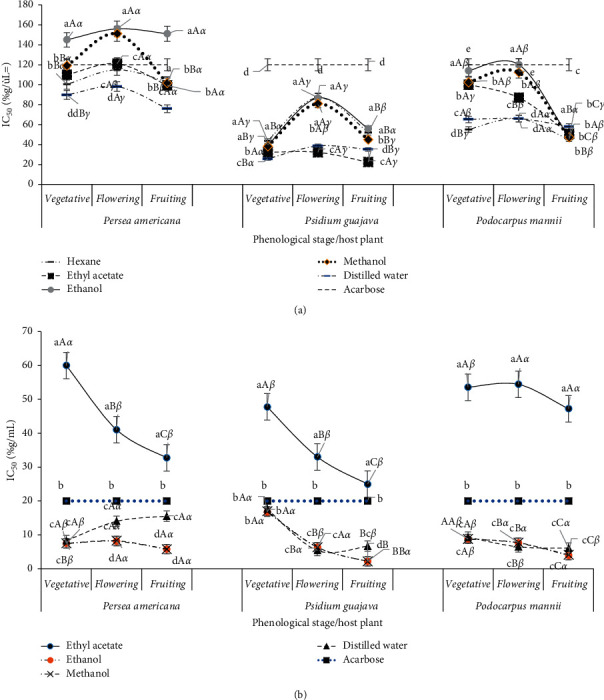
Evolution of *α*-amylase and *α*-glucosidase inhibition by *Phragmanthera capitata* extracts according to the phenological stage, host, and solvent of extraction. a, b, c, d, e: for the same phenological stage and host plant, means carrying different letters are significantly different (*p* < 0.05, Waller–Duncan test); A, B, C: for the same solvent and host plant, means carrying different letters are significantly different (*p* < 0.05, Waller–Duncan test); *α*, *β*, *γ*: for the same solvent and host phenological stage, means carrying different letters are significantly different (*p* < 0.05, Waller–Duncan test). (a) Inhibition of *α*-amylase. (b) Inhibition of *α*-glucosidase.

The influence of the solvent, phenological stage, and host on *α*-amylase and *α*-glucosidase inhibition by various extracts is presented in Figure 3. Extract from *P. capitata* collected on *P. americana* and *P*. *guajava* presented higher inhibitory activities (low IC_50_ compared to acarbose whose dose-activity is showed in Supplementary material) at vegetative and fruiting stages. With *P*. *mannii*, this inhibitory activity increased significantly during flowering and more at fruiting (Figure 3(a)). Ethyl acetate, methanol, and hexane extracts had the best inhibitory activity against *α*-amylase.

The hexane extract had poor inhibitory activity on *α*-glucosidase. The ethanol extract had a comparable inhibitory activity to that of the methanol extract. In general, regardless of the host and phenological stage, methanol, ethanol, and water extracts exhibited better inhibitory activities on *α*-glucosidase compared to the ethyl acetate extract.

### 3.4. Phenolic Contents of *Phragmanthera capitata*

**Figure 4:**
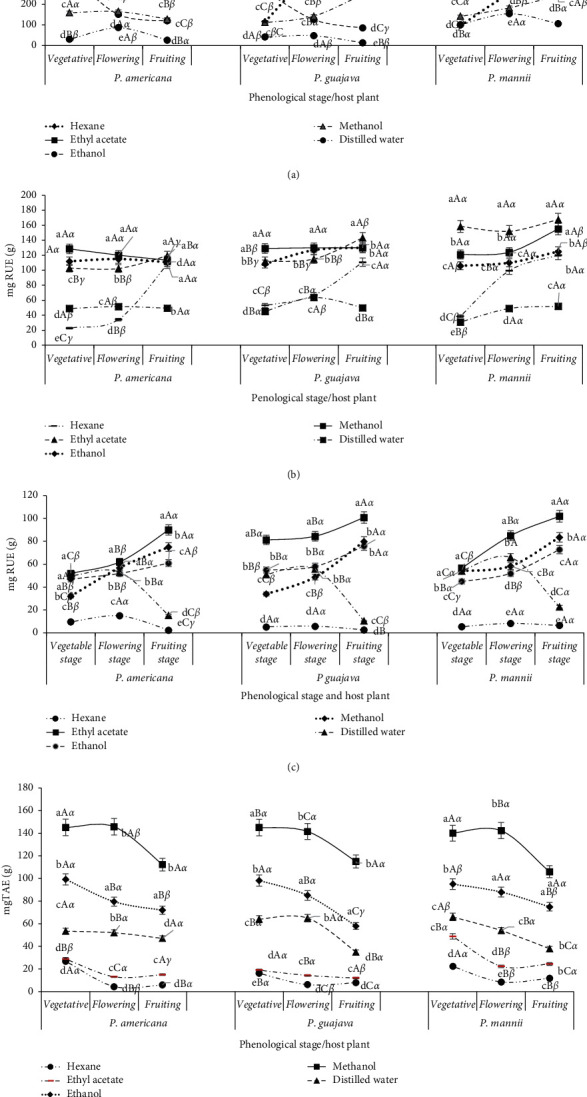
Evolution phenolic contents in *Phragmanthera capitata* extracts according to the phenological stage, host, and solvent of extraction. a, b, c, d, e: for the same phenological stage and host plant, means carrying different letters are significantly different (*p* < 0.05, Waller–Duncan test); A, B, C: for the same solvent and host plant, means carrying different letters are significantly different (*p* < 0.05, Waller–Duncan test), *α*, *β*, *γ*: for the same solvent and host phenological stage, means carrying different letters are significantly different (*p* < 0.05, Waller–Duncan test). (a) Total phenol contents. (b) Total flavonoid contents. (c) Total flavonol contents. (d) Total tannin contents.

The phenolics contains in extracts were determined using a standard calibration curve (Supplementary material). The evolution of the phenolic contents of *P. capitata* as a function of the solvent, host, and phenological stage is presented in Figure 4. The total phenol contents were significantly (*p* < 0.05) higher with ethyl acetate, followed by hexane at all stages of development of *P. capitata* and on all hosts (Figure 4(a)). Water was the worst solvent for extracting these compounds, while methanol and ethanol had globally comparable effects. With hexane and ethyl acetate, the total phenol contents increased significantly during flowering and during fruiting of *P. capitata* grown on *P. americana* and on *P. guajava*. With the other solvents, the general tendency was a decrease of the phenol content, the levels of total phenols being higher when *P. capitata* was harvested from *P. mannii*.

The total flavonoid contents of the extracts varied according to the extraction solvent, the phenological stage, and the host plant on which *P. capitata* grows (Figure 4(b)). Thus, this class of phenolic compounds was best extracted with methanol, ethanol, and ethyl acetate. Hexane and water extracted the lowest, regardless of the phenological stage and the host plant. However, for *P. capitata* collected on *P. mannii*, the total flavonoids were better extracted by ethyl acetate, and their contents were significantly different from those of methanol and ethanol extractions, respectively. Overall, the flavonoid levels of *P. capitata* were significantly higher during fruiting, especially when extracted with ethyl acetate and to a lesser extent ethanol and methanol regardless of the plant host on which *P. capitata* grows.

The solvents that enabled better extraction of total flavonols were, in the ascending order, ethyl acetate, methanol, and ethanol regardless of the phenological stage or the host plant (Figure 4(c)). For these three solvents, the flavonol contents increased significantly during flowering and even more during fruiting. Hexane was the solvent which extracted the flavonols the least, and for this solvent, the contents obtained were not influenced by the phenological stage and even less by the host plant. Overall, the water-extracted amounts of flavonols were practically comparable to ethanol, even though these quantities decreased significantly during fruiting. On the three hosts, the solvents behaved in the same way at all the phenological stages.

The solvent that best extracted the tannins from *P. capitata* was ethanol (Figure 4(d)), which exhibited significantly higher extraction capacity than methanol and water. With these three solvents, the rate of tannins decreased significantly during fruiting in all extracts, regardless of the host of *P. capitata*. With hexane and ethyl acetate, the tannin levels were low and decreased significantly during flowering except when the host was *P. mannii*, for which these levels remained constant during flowering and during fruiting.

### 3.5. Antioxidant Activities of *Phragmanthera capitata* Extracts

**Figure 5:**
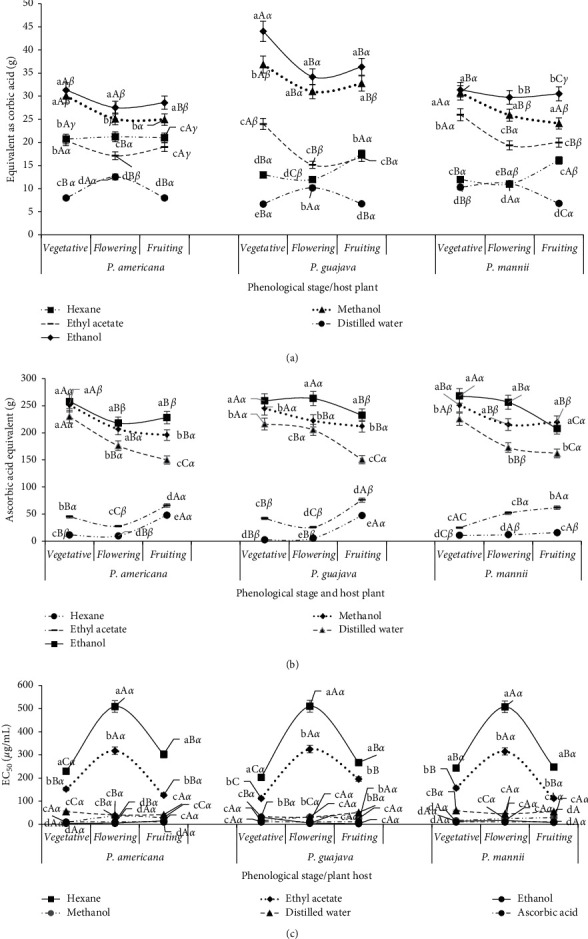
Evolution of antioxidant capacities of *Phragmanthera capitata* extracts according to the phenological stage, host, and solvent of extraction. a, b, c, d, e: for the same phenological stage and host plant, means carrying different letters are significantly different (*p* < 0.05, Waller–Duncan test); A, B, C: for the same solvent and host plant, means carrying different letters are significantly different (*p* < 0.05, Waller–Duncan test), *α*, *β*, *γ*: for the same solvent and host phenological stage, means carrying different letters are significantly different (*p* < 0.05, Waller–Duncan test). (a) Total antioxidant capacities. (b) FRAP activities. (c) DPPH.

The total antioxidant capacity, DPPH scavenging, and FRAP activities in various extracts of *P. capitata* varied according to the extraction solvent, the phenological stage, and the host plant (Figure 5). The total antioxidant capacity of the *P. capitata* extracts by various solvents decreased significantly in the following order: ethanol and methanol, ethyl acetate and hexane, and water at all stages of development of the plant and plant host (Figure 5(a)). The total antioxidant capacity decreased significantly during flowering regardless of the host plant and the extraction solvent, even though that of the aqueous extract increased during flowering. Furthermore, with ethanol and methanol, the total antioxidant capacity was significantly higher before and during fruiting of *P. capitata* growing on *P. guajava,* followed by that of *P. mannii*.

The solvents that gave extracts with better reducing power, in the ascending order were ethanol, methanol, and water regardless of the phenological stage or the host plant (Figure 4). Hexane and ethyl acetate gave extracts with worst reducing power (Figure 5(b)). For all solvents, the general trend was a decrease in the reducing power during flowering of *P. capitata* regardless of the host plant. On the other hand, with ethanol and methanol, the reducing power was higher before and during fruiting of *P. capitata* growing on *P. guajava* and on *P. mannii*.

Ethanol and methanol provided extracts with stronger DPPH scavenging activity, close to that of ascorbic acid regardless of the phenological stage and the host plant (Figure 5(c)). With hexane and ethyl acetate, the *P. capitata* extract had worst DPPH scavenging activity, and this activity was significantly weaker at the flowering stage of the plant. With water, DPPH scavenging activity of *P. capitata* extracts was intermediate to that of the extracts with solvents ethanol/methanol and ethyl acetate before and during flowering and significantly weaker during fruiting for all host plants. On the other hand, for all extracting solvents, DPPH scavenging activity of *P. capitata* extracts was not influenced by the host plant.

## 4. Discussion

The multiple biological activities and traditional use of *P*. *capitata* make this plant a good candidate for the preparation of a multiusage phytomedicine, especially against diabetes and oxidative stress, one of the factors that play a great role in the pathogenesis of diabetes [31]. However, for this to be effective, it is important to define the minimum conditions of its collection and preparation to guarantee its effectiveness at all times. This action goes through standardization to fulfill some basic requirements to guarantee phytomedicine efficacy, safety, and reproducibility. In this light, we studied some parameters that could influence the antidiabetic and antioxidant properties, the extraction yield, and the chemical profile of *P*. *capitata* extracts, including the nature of the extraction solvent, the host plant, and the phenological stage of *P*. *capitata* and observed that these factors had more or less marked effects on the studied properties.

Solid-liquid (plant powder-solvent) extraction was used in this study, for which the constituents of the plant are transferred to the solvent with a speed and efficiency which depended on the polarity, viscosity, and corrosive properties of the extracting solvent [30, 32]. In this light, the extraction yield of *P*. *capitata* was higher with polar solvents, namely, water, methanol, and ethanol, methanol being the best. Methanol, an amphiphilic solvent, with low viscosity and density, has the ability to weaken cellular structures, penetrate, and diffuse into the plant cell to solubilize a large range of molecules in the plant cells, mostly polar ones but also a large portion of nonpolar [31–34]. These properties may justify why this solvent gave better extraction yields than water. Our results are in accordance with those obtained for *P. capitata* growing on rubber in Nigeria [17].

Phenolic compounds belong to a large family of secondary metabolites and possess different chemical structures and polarities and are differently extracted by various solvents [35]. The difference in extractive capacities of solvents on phenolic compounds of *P. capitata* is in agreement with the findings of Ohikhena et al. [17] and Freitas et al. [36]. The global trend was a decrease in the total phenol, flavonoid, and flavonol contents with the increase of solvent polarity, particularly with methanol. For this solvent, the extraction yield was significantly and negatively correlated to TPC (*r* = 0.877, *p* < 0.01), TFC (*r* = −0504, *p* < 0.01), and TFnC (*r* = −701, *p* < 0.01), while TTC was positively correlated to the extraction yield (*r* = 0.572, *p* < 0.01) by this solvent (Table 2). This can be justified by the fact that, in methanol extracts, these compounds were surely diluted, due to the high and nonspecific extractive capacity of plant secondary metabolites by methanol, allowing it to extract a large variety of compounds, both polar and nonpolar [37]. The positive correlation of the yield to TTC indicates that tannins were more efficiently extracted by methanol. Indeed, tannins are polymerized into complex forms of phenolic compounds with increased polarity [38]. The yield was positively correlated to TFC (*r* = 0.868; *p* < 0.01) and TTC (*r* = 0.529; *p* < 0.05) of ethyl acetate extract (Table 2), suggesting that theses extracts mostly contain aglycone flavonoids. It is known that less polar solvents such as ethyl acetate extract preferably extract aglycone flavonoids, while more polar solvents such as alcohol and water better extract glycoside flavonoids [39]. These correlations, associated to one of the methanol extractions, may indicate that the total tannins of *P. capitata* are midpolar compounds. However, it is to be noted that many authors recommend solvent mixtures for tannin extraction [40], and this may later be experimented for *P. capitata* tannin extraction, combining, for example, methanol and ethyl acetate in appropriate proportions.

Plants contain a wide range of active principle described as having hypoglycemic effects, mainly glycosides, alkaloids, hypoglycans, galactomannan gum, polysaccharides, steroids, peptidoglycan, guanidine, glycol peptides, and terpenoids [41]. Some of these principles are *α*-amylase inhibitors, and many studies have focused their attention on phenolic compounds as amylase inhibitors [42] and as antioxidant [43]. *α*-amylase and *α*-glucosidase are a key enzyme in digestion of starch, and as such, their inhibitors such as acarbose are used to control postprandial glycemic in type-2 diabetic [44]. Recent studies show a renewed interest in plant phenolic compounds as inhibitors of *α*-amylase and *α*-glucosidase [45]. In the present study, the inhibition of *α*-amylase was negatively correlated to yield for hexane (*r* = −0.452, *p* < 0.05), ethanol (*r* = −0.497, *p* < 0.01), and methanol (*r* = −0.487, *p* < 0.01) extracts, indicating that high yield is associated to low IC_50_ and consequently high inhibition (Table 2). The inhibitory capacity of the hexane extract was not correlated to any phenolic content, a suggestion that the active principles in these extracts are not phenolic compounds. This was not the case with glucosidase that reveals a positive correlation between extraction yield and enzyme inhibition for methanol extracts (*r* = 0.485, *p* < 0.05). In contrast, for this extract, a negative correlation was observed between IC_50_ of *α*-glucosidase (*r* = -0.694, *p* < 0.01) and TfnC (Table 3). For the ethyl acetate extracts, the inhibitions of *α*-amylase and *α*-glucosidase were negatively correlated with TPC (*r* = −0.433, *p* < 0.05) and TfnC (*r* = −0.496, *p* < 0.01), suggesting that the active ingredients in these extracts could be flavonols. With ethanol extracts, the correlation between enzyme inhibition was negative for TFC (*r* = -0.602, *p* < 0.01) and TfnC (−0,672, *p* < 0.01), suggesting that the active principles could be distributed in these two groups of phenolic compounds. With the methanol extract, a negative correlation was highlighted between TPC (*r* = −0.735, *p* < 0.01) and TfnC (*r* = −0.746, *p* < 0.01) and the inhibition of *α*-glucosidase, whereas for *α*-amylase, this correlation exists with TTC (*r* = −0.616, *p* < 0.01). For the aqueous extract, no correlation was highlighted.

With ethanol and methanol extracts, inhibition of *α*-amylase was positively correlated with FRAP (*r* = 0.486 and 0.528, respectively, *p* < 0.01), showing that the active compounds could also have ferric reducing ability. According to Asgar [44], it is important to keep equilibrium between *α*-amylase and *α*-glucosidase inhibitors, which efficiently limits the gastrointestinal adverse effects related to undigested starch reaching the colon. *P. capitata* extract that inhibited both *α*-amylase and *α*-glucosidase (except hexane extract) responding to this need. With lower IC_50s_, the activities of the extracts on *α*-glucosidase were more pronounced, and this has varied with the extraction solvent. IC_50_ of methanol and ethyl acetate extracts were negatively correlated to these properties for TPC and TfnC. This shows that the compounds which inhibit *α*-glucosidase are different from those which inhibit *α*-amylase and are mainly effective in the flavonol group. *P. capitata* growing on rubber tree in Nigeria showed a weak *α*-amylase inhibition across all the solvent extracts and stronger *α*-glucosidase inhibition like our results [18]. The weak *α*-amylase inhibition by *P. capitata* grown on rubber in the previous study compared to our results may show the influence of the host plant and, to a lesser extent, the geographical conditions, and thus the importance of standardization.

As a mistletoe, *P. capitata* is capable of penetrating the living tissue of the host plant's stems and branches to extract the necessary resources for their survival. For this purpose, this parasitic plant uses haustorium induced by host-derived phenolic compounds to penetrate host tissues [46]. It depends on its host for water, nutrients, and some carbon compounds. It is also suggested that active compounds may pass from the host trees to the parasitic plants [1, 47]. On the other hand, the parasitic plant is able to synthesize its own secondary metabolites to fight against the resistance of the host [48]. This bilateral relationship may justify the differences in composition of phenolic compounds, and therefore the difference in the inhibition of glycolytic enzymes observed about the three host plants, which have different physiological and photosynthetic behaviors, and they differently react to infestation by *P. capitata*. This is in accordance with the results of [47, 49] who showed that *Viscum album* (European mistletoe) exhibited different levels of total phenolic acids and antioxidant activity when parasitizing different host species. In addition, it is important to mention that Dibong et al. [50] showed that *Persea americana*, with 9.38% abundance and 21.51% parasitism, is more susceptible to *P. capitata* than *P*. *guajava* with 4.12% abundance and 35% parasitism in Cameroun. This shows that the two plants possess two different mechanisms of defense that can justify the qualitative and quantitative differences in phenolic contents and activities of *P*. *capitata* collected on these two host plants.

The levels of secondary metabolites in plants can vary depending on the environmental conditions and the stage of development of the plant [51]. Phenolic compounds, which are one of the main groups of secondary metabolites, are not an exception. In the present study, it became clear that the total phenol contents increased significantly from the vegetative stage to the flowering stage and from the flowering stage to the fruiting stage. These results concur with those of Berezina et al. [52] who showed that the rate of production of phenolic compounds is lower in *Vaccinium macrocarpon* plants during budding compared to flowering and fruiting stages. The increase in the total phenolic content during flowering may be attributed to flavonoids, which are responsible for the coloring of pollen and petals of flowers and which in some cases represent up to 4% of the dry weight of these flowers [53], since, in the present study, the evolution of flavonoids levels followed that of total phenols with a few exceptions. This is in agreement with the results of Medini et al. [54] which revealed that the total phenol content of *Limonium densiflorum* increased at the flowering stage as compared to the vegetative one. Flowering and fruiting of *P*. *capitata* occur during the raining season. Thus, both the hosts and *P*. *capitata* are under favorable photosynthetic conditions (sunshine, abundance of minerals, and water) [51], boosting the rate of secondary metabolite biosynthesis in these plants [52]. At the same time, in this study, the rate of production of total tannins decreased from the vegetative phase to the fruiting stage, certainly passing to flowers. The main function of tannins is not only to ensure the protection of the plant against microbial pathogens, harmful insects, and other herbivores but also allow the reinforcement of plant tissues [55]. Plants rich in phenolics can also negatively modify the growth of neighboring plants by limiting the nitrogen supply. It can therefore be estimated that during the flowering of the fruiting body, this defense system in *P*. *capitata* works less intensely.

## 5. Conclusion

In this study, the effect of the extraction solvent, host plant, and phenological stage on phytochemical compounds, antioxidant, and antidiabetic activities of *Phragmanthera capitata* were investigated. Our results show that the extraction solvent, host, and phenological stages significantly affect the phenolic content, antioxidant, and antidiabetic activities of *P. capitata*. Out of the five solvents used for extraction of phytochemical compounds from the vegetable material, ethyl acetate was the most suitable solvent for optimum recovery of the phenolic, flavonoid, and flavonol compounds of *P. capitata*; while ethanol and methanol were the most suitable for tannins. Among host plants, the total phenolic, flavonoid, flavonol, and tannin contents of *P. capitata* extract from *Persea americana* and *Psidium guajava* were significantly higher than those of *P. capitata* extract from *Podocarpus mannii*. Better total antioxidant and antidiabetic activities were found with *P. capitata* growing on *P. americana* and *Psidium guajava*. Moreover, both phenolic compounds and antioxidant and antidiabetic activities of *P. capitata* appeared higher during flowering and fruiting. The HPLC profile of the ethyl acetate extract showed that it contains gallic acid, quercetin, rutin, and tannic acid, which could be related to the biological properties of the plant. The phytochemical composition and HPLC profile revealed the dynamic accumulation of phenolic compounds and established a basis for determining the best solvent and phenological stage for harvesting of the plant to improve the content of beneficial compounds in the phytomedicine from *P. capitata*.

## Figures and Tables

**Table 1:** Information about the plant material used in the study.

Species	Role	Registered voucher code
*Phragmanthera capitata* (Sprengel) S. Balle	Studied plant	24673/SRF/CAM
Persea americana Mill. (Lauraceae)	Host 1	57756/HNC
*Psidium guajava* Linn. (Myrtaceae)	Host 2	2884/SRF/CAM
*Podocarpus mannii* Hook. f (Podocarpaceae)	Host 3	5088/HNC

SRF, Cameroon forest reserve society; HNC, Cameroon national herbarium.

**Table 2:** Significant Pearson correlation coefficients between yield and *α*-amylase and *α*-glucosidase inhibition, phenolic contents, and antioxidant activities of various extracts from *Phragmanthera capitata* as a function of solvent of extraction (regardless the host plant).

Phenolic contents					Antioxidant properties	Enzyme inhibition	
Extracts	TPC	TFC	TfnC	TTC	FRAP	*α*-amylase	*α*-glucosidase
Hexane		−**0.573**^*∗∗*^		**0.385** ^*∗*^	−**0.558**^*∗∗*^	−**0.452**^*∗*^	
Ethyl acetate		**0.868** ^*∗∗*^		**0.529** ^*∗∗*^			
Ethanol					**0.384** ^*∗*^	−**0.497**^*∗∗*^	
Methanol	−**0.877**^*∗∗*^	−**0.504**^*∗∗*^	−**0.701**^*∗∗*^	**0.572** ^*∗∗*^		−**0.487**^*∗*^	**0.485** ^*∗*^
Aqueous	**0.645** ^*∗∗*^						

^*∗*^ *p* < 0.05. ^*∗∗*^*p* < 0.01.

**Table 3:** Significant Pearson correlation coefficients between phenolic contents, antioxidant activities, and *α*-amylase and *α*-glucosidase inhibition according to extraction solvent (regardless the host plant).

		Phenolic contents			Antioxidant activities			Enzyme inhibition		
Solvent		TPC	TFC	TfnC	TTC	TAC	FRAP	DPPH	Amylase	Glucosidase
Hexane	FRAP	0.591^*∗∗*^	0.622^*∗∗*^	−0.571^*∗∗*^		0.476^*∗*^				
	DPPH			0.487^*∗∗*^	−0.658^*∗∗*^					

Ethyl acetate	FRAP	0.527^*∗∗*^		0.714^*∗∗*^	−0.374			−0.418^*∗*^	−0.561^*∗∗*^	−0.506^*∗∗*^
	DPPH					−0.608^*∗∗*^	−0.418^*∗*^			
	Amylase	−0.433^*∗*^		−0.771^*∗∗*^	0.827^*∗∗*^	0.591^*∗∗*^	−0.561^*∗∗*^			0.581^*∗∗*^
	Glucosidase	−0.496^*∗∗*^		−0.611^*∗∗*^	0.626^*∗∗*^	0.572^*∗∗*^	−0.506^*∗∗*^		0.581^*∗∗*^	

Ethanol	FRAP	0.466^*∗*^	−0.414^*∗*^	−0.643^*∗∗*^	0.701^*∗∗*^	0.393^*∗*^				0.486^*∗*^
	DPPH		−0.644^*∗∗*^		0.450^*∗*^	0.532^*∗∗*^				0.815^*∗∗*^
	Amylase	0.555^*∗∗*^	−0.602^*∗∗*^	−0.691^*∗∗*^	0.432^*∗*^	−0.691^*∗∗*^				
	Glucosidase	0.466^*∗*^	−0.672^*∗∗*^	−0.577^*∗∗*^	0.747^*∗∗*^	0.566^*∗∗*^	0.486^*∗*^	0.815^*∗∗*^		

Methanol	FRAP		0.157	−0.709^*∗∗*^	0.517^*∗∗*^	0.603^*∗∗*^		−0.854^*∗∗*^	0.104	0.528^*∗∗*^
	DPPH		0.045	0.630^*∗∗*^	−0.360	−0.576^*∗∗*^	−0.854^*∗∗*^		−0.098	−0.462^*∗*^
	Amylase	0.658^*∗∗*^	0.454^*∗*^	0.510^*∗∗*^	−0.616^*∗∗*^	0.009	0.104	−0.098		−0.694^*∗∗*^
	Glucosidase	−0.735^*∗∗*^	−0.232	−0.746^*∗∗*^	0.646^*∗∗*^	0.526^*∗∗*^	0.528^*∗∗*^	−0.462^*∗*^	−0.694^*∗∗*^	

Water	FRAP			0.678^*∗∗*^	0.802^*∗∗*^					
	DPPH		−0.553^*∗∗*^		−0.397^*∗*^					−0.418^*∗*^
	Amylase									−0.573^*∗∗*^
	Glucosidase							−0.418^*∗*^	−0.573^*∗∗*^	

^*∗*^ *p* < 0.05. ^*∗∗*^*p* < 0.01.

## Data Availability

The data used to support this study are made available from the corresponding author upon request.

## Abbreviations

TPC: Total phenols content
TFC: Total flavonoids content
TfnC: Total flavonols content
TTC: Total tannins content
TAC: Total antioxidant capacity
RUE: Rutin equivalent
TAE: Tannic acid equivalent
AUC: Area under curve
DPPH: 2,2-Diphenyl-1-picrylhydrazyl
GAE: Gallic acid equivalent
FRAP: Ferric ion reducing antioxidant power
HPLC: High-performance liquid chromatography
DMSO: Dimethyl sulfoxide
DNSA: 3,5-Dinitrosalicylic acid
RSA: Radical scavenging activity.

## Supplementary Materials

Supplementary Materials Dose activities of acarbose and calibration curves of standards for phenolics content determination. Isolated compounds from the ethanol extract of *Phragmanthera capitata* collected during fruiting on *Persea americana*.
