# Supplementary material for: The Influence of Solvent, Host, and Phenological Stage on the Yield, Chemical Composition, and Antidiabetic and Antioxidant Properties of Phragmanthera capitata (Sprengel) S. Balle
Source: Evid Based Complement Alternat Med. 2020 Nov 18;2020:6284925. doi: 10.1155/2020/6284925 (PMC7691007; doi:10.1155/2020/6284925)
Supplement: Supplementary Materials — Dose activities of acarbose and calibration curves of standards for phenolics content determination. Isolated compounds from the ethanol extract of Phragmanthera capitata collected during fruiting on Persea americana. [file 6284925.f1.docx]

**Dose-response curves between standard compounds and activities (enzymes and DPPH-RSA)**

**Dose-response between acarbose concentration and α-amylase inhibition**

**Dose-response between acarbose concentration and α-glucosidase inhibition**

**Dose-response between ascorbic acid concentration and DPPH radical inhibition**

**Calibration curves for phenolics content and antioxidant determination**

Gallic acid calibration curve for total phenol content determination

Rutin calibration curve for total flavonoid content determination

**Rutin calibration curve for total flavonol content determination**

Tannic acid calibration curve for total tannin content determination

**Ascorbic acid calibration curve for Total antioxidant capacity determination**

**Ascorbic acid calibration curve for FRAP determination**

**Isolated compounds from ethanol extract of *Phragmenthera capitata* collected during fruiting on *Persea americana***

Part of the ethanol extract (52g) was subjected to column chromatography over silica gel (0.2-0.5 mm) using successively Hex/ EtOAc and EtOAc/MeOH by increasing polarity. The fractions were recovered, evaporated under reduced pressure and were gathered on the basis of their TLC profiles leading to three major fractions (A-C). Fraction A (10.7 g) was subjected to a CC over silica gel (0.2-0.5 mm) using Hex/EtOAc (9:1, 8:2, 7:3) to afford lupeol (**1**, 20.9 mg) and 71.4 mg of a mixture of stigmasterol (**2**) and *β*-sitosterol (**3**). Fraction B (8.7 g) precipitated partially in acetone to give a yellow powder. The filtration and purification of this powder using Sephadex LH-20 (CH_2_Cl_2_/ MeOH 1:1) afforded quercetin (**4**, 18.2 mg)*.* Fraction C (24.2 g) after filtration was subjected to a CC over silica gel (0.2-0.5 mm) using EtOAc/MeOH (9.5:0.5, 9:1, 8:2) to afford *β*-sitosterol 3-*O-β*-D-glucopyranoside (**5**).

The structures of isolated compounds were established from analysis of their
spectroscopic data (IR, ^1^H and ^13^C NMR) and comparison with those from literature. NMR spectra were performed on a 500 MHz frequency JOEL spectrometer. The chemical shifts (δ) are expressed in parts per million (ppm) with TMS (Me_4_Si, δ=0) as an internal reference and the values ​​of the coupling constants (J) are expressed in Hertz (Hz). IR spectra were recorded on a SHIMADZU spectrometer.

Lupeol (**1**), Stigmasterol (**2**), *β*-sitosterol (**3**), Quercetin (**4**), *β*-sitosterol 3-*O*-*β*-D-glucopyranoside (**5**)
